# The evaluation of left ventricular function for patients being considered for, or receiving Trastuzumab (Herceptin) therapy

**DOI:** 10.1038/sj.bjc.6603340

**Published:** 2006-10-24

**Authors:** K F Fox

**Affiliations:** 1 Department of Cardiology, Hammersmith Hospitals NHS Trust, BSE Administration, 10 Greycoat Place, London Victoria SW1P 1SB, UK

**Sir**,

The British Society of Echocardiography, whose 2400 members represent physiologists (technicians) and clinicians performing echocardiography in the UK, has welcomed the recent NICE guidelines on the use of Trastuzumab.

We are aware of the importance of monitoring left ventricular (LV) function and believe echocardiography and BSE accredited echocardiogrpahers are ideally placed to perform this monitoring.

We have issued a statement (also available at www.bsecho.org under ‘Guidelines’) to our members, which, we also believe will be of importance and interest to Oncologists.

*British Society of Echocardiography – Statement to Members*

*The evaluation of left ventricular function for patients being considered or receiving Trastuzumab (Herceptin) therapy*

1. The NICE have recently extended their guidance on indications for Trastuzumab. As part of the care of patients considered for Trastuzumab pre-treatment measurement of LV function is required and 3 monthly during treatment according to most protocols. This is because of the known potential for cardiac damage occurring in patients receiving Trastuzumab.
2. Although local guidelines may vary, current guidelines typically state that patients should not normally be commenced on Trastuzumab if their baseline Ejection Fraction (EF) is ⩽55%. If the EF falls by more than 10% or to <50% cessation of treatment should be considered.
3. Echocardiography is an ideal technique for the evaluation of LV function. A small number of patients, usually owing to local surgery, may have unacceptably poor images and should be considered for an alternative imaging modality.
4. Ejection Fraction can be disputed as the optimal method of describing LV function but it has been chosen in considerations regarding Trastuzumab therapy.
5. The exacting nature of the guidelines require accurate measurement of EF. This is likely to be only possible by the use of (biplane) Simpson's Rule Method or 3D echocardiography, with the availability of contrast for LV opacification, by appropriately skilled echocardiographers.
6. Echo laboratories performing such studies should have evidence (e.g. through audit or other Quality Control processes), not more than 12 months old, that they can reproducibly measure EF to the requirements of the guidelines. This means that they can identify a 10% change in EF as a true change.
7. Cardiology departments and oncology departments should cooperate closely in individual (borderline) cases and in the provision of the echo service.
8. It is unreasonable to expect already hard pressed echo laboratories to absorb a significant increase in workload relating to Trastuzumab therapy without consideration of the provision of appropriate resources (staff, machine time, and funds). This should be resolved locally.

The Council of the British Society of Echocardiography, June 2006.

